# Stenosis quantification in high-pitch photon-counting coronary CT angiography: *in vitro* and *in vivo* impact of reconstruction kernel types and sharpness levels

**DOI:** 10.1186/s41747-025-00635-5

**Published:** 2025-09-24

**Authors:** Jonathan Stock, Mortiz Halfmann, Tilman Emrich, Lukas Müller, Nicola Fink, Dirk Graafen, Tobias Bäuerle, Michaela Hell, Martin Geyer, Milan Vecsey-Nagy, Akos Varga-Szemes, Yang Yang

**Affiliations:** 1 Department of Radiology, Ludwig Maximilian University Hospital, Munich, Germany; 2 Department of Diagnostic and Interventional Radiology, University Medical Center Mainz, Mainz, Germany; 3 Division of Cardiovascular Imaging, Department of Radiology and Radiological Science, Medical University of South Carolina, Charleston, SC USA; 4 Deutsches Zentrum für Herz-Kreislauf-Forschung (DZHK), Berlin, Germany; 5 Department of Cardiology, University Medical Center Mainz, Mainz, Germany

**Keywords:** Computed tomography angiography, Coronary stenosis, Phantoms (imaging), Reproducibility of results, Tomography (x-ray computed)

## Abstract

**Background:**

We investigated the influence of different kernel types and sharpness levels on *in vitro* and *in vivo* coronary stenosis quantification in high-pitch photon-counting detector coronary CT angiography (PCD-CCTA).

**Materials and methods:**

Coronary stenoses were evaluated in a phantom containing two stenosis grades (25% and 50%), and in a retrospective cohort of 30 patients who underwent high-pitch PCD-CCTA. Scans were reconstructed as virtual monoenergetic images at 55 keV using three different kernels (Br, Bv, and Qr) and four sharpness levels (36, 40, 44, and 48). Percent diameter stenosis (PDS) values were compared. *In vitro* measurements were additionally compared with the stenosis reference value. Two readers independently assessed the *in vivo* measurements.

**Results:**

*In vitro*, PDS values of all stenoses showed no difference among various kernel types and sharpness levels (*p* ≥ 0.412). However, PDS measurements using kernel Bv40 showed the smallest cumulative deviation from the ground truth. *In vivo*, a total of 53 stenoses were identified in 30 patients, aged 63 ± 13 years (mean ± standard deviation), 8/30 (27%) females. There was no significant difference in PDS measurements among reconstructions, either when analyzed per stenosis or stratified by different plaque types (*p* = 1.000). Bv kernels showed higher interobserver reliability (intraclass correlation coefficient: Bv 0.91; Qr 0.88; Br 0.85).

**Conclusion:**

With comparable diagnostic accuracy, different kernel types and sharpness levels can be used in high-pitch PCD-CCTA. Due to the *in vivo* advantage in interobserver reliability and the *in vitro* observed lowest cumulative deviation from ground truth, reconstruction with kernel Bv40 should be preferred.

**Relevance statement:**

For image reconstruction in PCD-CCTA with high-pitch mode, kernel Bv40 should be considered to obtain the best diagnostic performance and reliability of stenosis quantification.

**Key Points:**

- High-pitch PCD-CCTA images can be reconstructed with different kernels.
- Reconstructions with different kernels showed comparable accuracy on coronary stenosis quantification.
- *In vitro*, Bv40 reconstructions showed superior measurement accuracy to the reference.
- *In vivo*, reconstructions with the Bv kernel had the highest interobserver reliability.
- Reconstruction with kernel Bv40 should be considered in high-pitch PCD-CCTA.

**Graphical Abstract:**

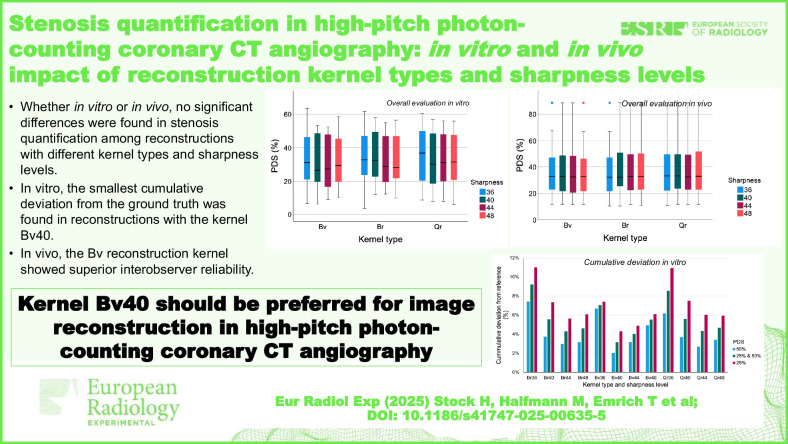

## Background

Coronary artery disease (CAD) is the most common heart condition and the leading cause of mortality worldwide [1]. Over the past 20 years, coronary computed tomography angiography (CCTA) has become a well-established noninvasive method for the diagnostic work-up of patients with suspected stable CAD. Due to its excellent sensitivity and high negative predictive value, CCTA is recommended by major international guidelines as the first-line test to rule out obstructive CAD, especially in patients with low to intermediate risk [2–4].

Recently, the first-generation dual-source photon-counting detector (PCD) computed tomography (CT) was introduced and approved for routine clinical use. Compared with conventional energy-integrating detector CT, PCD-CT possesses several advantages, including higher spatial resolution, increased contrast-to-noise ratio, and superior dose efficiency [5–9]. It has the potential to significantly improve the image quality and expand the utility of cardiovascular imaging [10–15].

Advances in PCD-CT reconstruction algorithms are also expected to enhance the accuracy of coronary artery plaque and stenosis quantification [16, 17]. Previous studies investigated the influence of reconstruction kernels and sharpness levels on image quality in high-pitch CCTA [18] and ultra-high-resolution (UHR) CCTA with PCD-CT [19]. *In vitro* and *in vivo* studies indicated that sharper vascular reconstruction kernels improve coronary stenosis quantification and plaque characterization in UHR PCD-CCTA [16, 20]. However, the effects of reconstruction kernels and sharpness levels on stenosis assessment in high-pitch PCD-CCTA, the less dose-intensive alternative to UHR PCD-CCTA for patients with lower pretest probability of CAD, remain unknown.

Therefore, the aim of this study was to investigate the influence of different kernel types and sharpness levels on coronary artery stenosis quantification in high-pitch PCD-CCTA.

## Materials and methods

### Dynamic vessel phantom

**Fig. 1:**
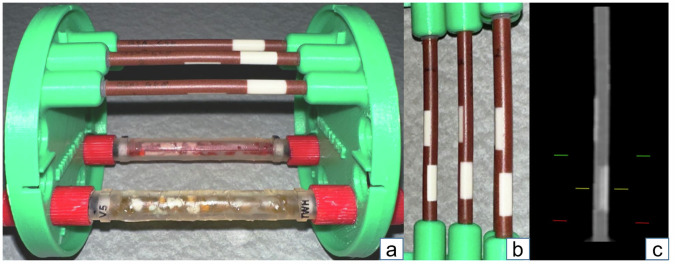
Custom-made vessel phantom. **a** An assortment of vessel phantoms in a three-dimensional-printed mounting. **b** From left to right: phantom vessel contains 1,000-HU mixed plaques, 1,000-HU calcified plaques, and 800-HU calcified plaques. **c** PCD CT scan of one vessel phantom in coronal reconstruction

**Fig. 2:**
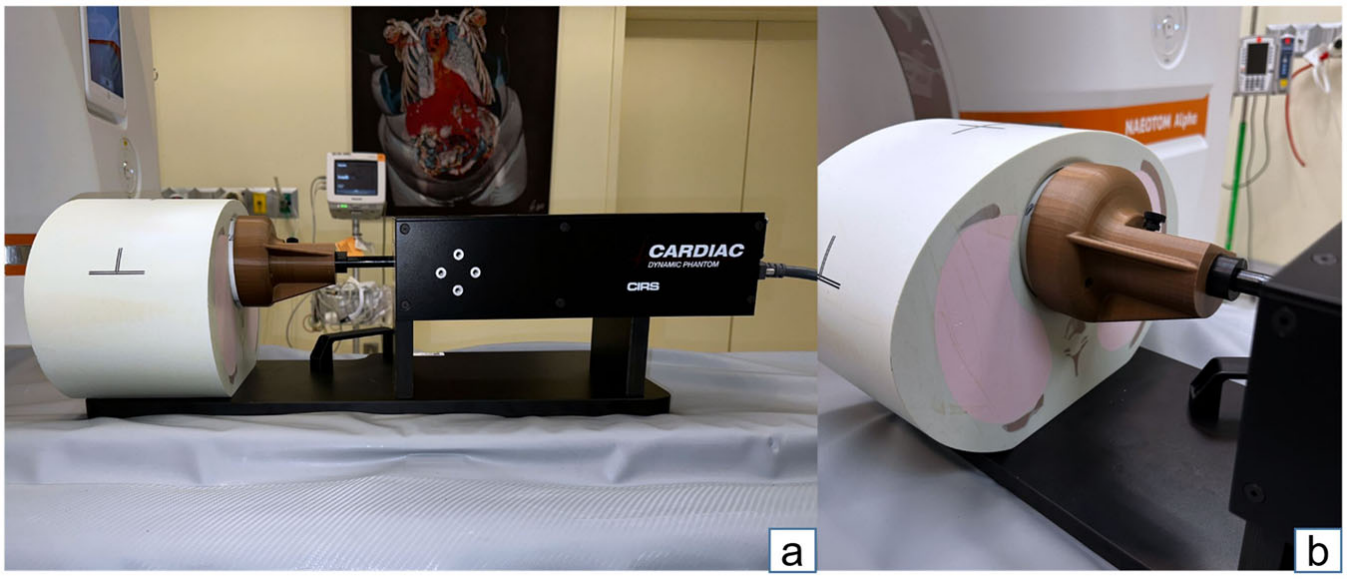
Dynamic chest phantom. **a** The dynamic chest phantom (CIRS Dynamic Cardiac Phantom, Model 008) comprises an anthropomorphic thorax CT body, moving rod, motion actuator, motion controller, and CIRS Motion Control software. The phantom body contains a fully articulated spine, ribs, and lungs in order to represent the average human thorax in shape, proportion, and composition. **b** A three-dimensional-printed mount affixes the rod that inserts into the thorax phantom and houses the vessel phantoms

The dynamic vessel phantom included a rod that contained three custom-made vessels with a diameter of 4 mm. The vessels were composed of solid material, mimicking a physiological mixture of iodine contrast medium and blood with an attenuation of 800 HU in one vessel and 1,000 HU in the other two vessels. Each vessel contained two artificial plaques simulating percentage diameter stenosis (PDS) of 25% and 50%. One vessel with 1,000 HU contained mixed plaques, and the other two vessels with 1,000 HU and 800 HU contained calcified plaques (Fig. 1). The rod was mounted in an anthropomorphic chest CT phantom (Fig. 2), which has been described in prior studies [21, 22].

### Phantom CT data acquisition

Phantom scans were performed on a first-generation, dual-source CT system (NAEOTOM Alpha, Siemens Healthineers), which contains two photon-counting cadmium telluride detectors with a collimation of 144 × 0.4 mm. The phantom acquisitions used a tube voltage of 120 kVp, a tube current-time product of 100 mAs, a field of view of 200 × 200 mm, and a matrix size of 512 × 512 pixels.

During the image acquisition, heart motion was simulated using a 4D coronary motion simulator with a 10-cm diameter water carrier (Cardio CT Phantom and Sim4DCardio, QRM). In combination with an artificial electrocardiographic signal, scanning was performed at 60, 80, and 100 beats per minute with electrocardiographic triggering in the diastole, starting at 70% R–R interval. The phantom scans were repeated five separate times at each heart rate and once more without simulated cardiac motion.

### Patient cohort

This retrospective study was approved by the institutional ethics committee (reference number: 2022-16359) with waivers of informed consent and performed in compliance with the Declaration of Helsinki.

Patients who underwent CCTA between February and March 2022 were retrospectively identified based on the following inclusion criteria: (1) CCTA was indicated to exclude or determine CAD, and (2) CCTA was performed with high-pitch “Flash” mode on PCD-CT. Exclusion criteria were non-evaluable coronary arteries or contraindication to nitroglycerin.

### Patient CT data acquisition

Patient scans were performed on the same dual-source PCD system. In the absence of contraindications, patients were administered 0.8 mg sublingual nitroglycerin and a maximum of 10 mg intravenous metoprolol depending on the heart rate. All patients received a dual-bolus injection consisting of 15 mL iodinated contrast agent (Ultravist 370 mgI/mL, Bayer Healthcare) for the test bolus and 70 mL for the main bolus, followed by a saline chaser (25 mL). The electrocardiographic pulsing window was set in the diastole with a start at 60% of the R–R interval. CT scans were acquired at 120 kVp with automated tube current modulation (CARE Dose4D, Siemens Healthineers) in the “Flash-QuantumPlus” mode (pitch factor 3.2, gantry rotation time 0.25 s) with a detector collimation of 144 × 0.4 mm.

### Image reconstruction

*In vitro* reconstructions were performed using a proprietary offline raw data reconstruction platform (ReconCT version 15.0.57554.0, Siemens Healthineers), while *in vivo* reconstructions were processed directly on the PCD-CT system.

*In vitro* and *in vivo* CT images were reconstructed using three different kernels (Body regular [Br], Body vascular [Bv], and Quantitative regular [Qr]) and four sharpness levels (36, 40, 44, and 48), resulting in a total of 12 reconstructions per CT scan. The image slice thickness was set to 0.4 mm with an increment of 0.2 mm. The vendor-specific level of iterative reconstruction (Quantum iterative reconstruction) was set at the maximum available level (level 3) at the time of the investigation. Virtual monoenergetic images were reconstructed at 55 keV.

### Stenosis assessment

*In vitro* scans were evaluated by a trained reader (J.S.) with 2 years of experience in cardiovascular imaging, under close supervision by a board-certified cardiovascular radiologist (T.E.) with more than 13 years of experience in the field. The analysis of *in vivo* scans was assessed by two readers, one (M.H.) with 5 years of experience and one board-certified cardiovascular radiologist (Y.Y.) with 10 years of experience in cardiovascular imaging.

Stenosis assessment was performed using a commercially available software solution (Syngo.via, Version VB60A, Siemens Healthineers). The CT scans were presented anonymized without image information and in a random order to minimize recall bias. Readers were blinded to kernel, sharpness level, and each other’s results. Standard window setting was used with the window level corresponding to the attenuation in the aorta at the level of the left main coronary artery and the window width obtained by multiplying the attenuation by 2.5 [19].

**Fig. 3:**
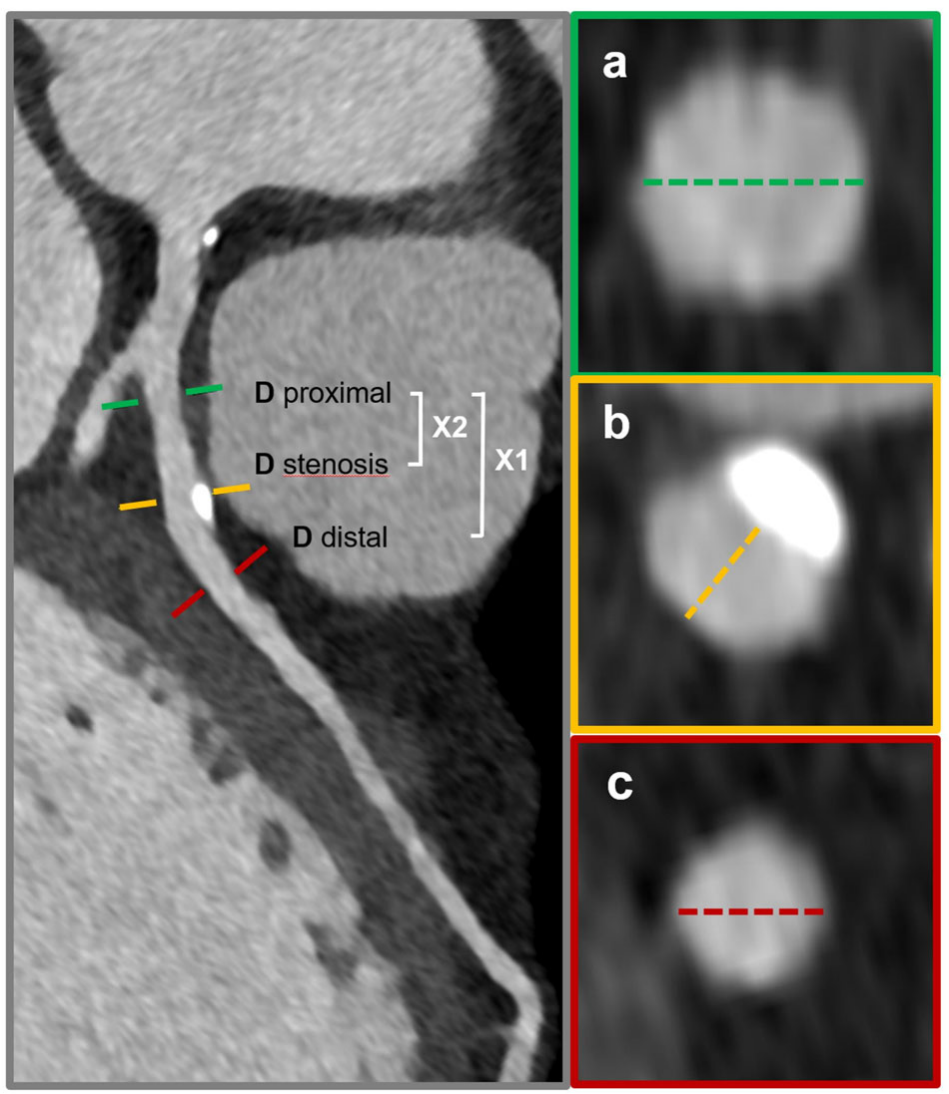
Assessment of coronary stenosis *in vivo*. Curved multiplanar reconstruction (figure on the left) shows a calcified plaque with proximal left coronary artery narrowing. Vessel diameter at the level of stenosis (**b**, orange line), as well as at the level of the closest unaffected vessel proximal (**a**, green line) and distal to the stenosis (**c**, red line), was measured. The distances along the centerline between the proximal and distal reference sites (between the green and red level, X1) and between the proximal reference site and stenosis site (between the green and orange level, X2) were determined

Stenosis quantification was performed on cross-sectional images with vessel diameters measured perpendicular to the vessel centerline. PDS was calculated *in vitro* using the following formula [23]:

$${{{\rm{PDS}}}}=1-\frac{{{\it{D}}}_{{{{\rm{stenosis}}}}}}{{{\it{D}}}_{{{{\rm{ref}}}}}}$$

where *D*_stenosis_ is the minimal lumen diameter within the stenosis and *D*_ref_ is the average of diameters at the proximal and distal reference points, which were placed at the nearest areas without coronary plaques or wall abnormalities. Since this formula was developed and used for phantom studies with constant vessel diameter and equal distance between stenosis and reference points, a modified formula from Cheng et al, with consideration of the physiological vessel narrowing from proximal to distal segments, was used for *in vivo* stenosis assessment [24]. Accordingly, *in vivo* PDS was calculated using the following formula:

$${{{\rm{PDS}}}}=1-\frac{{{\it{D}}}_{{{\rm{stenosis}}}}}{{{\it{D}}}_{{{\rm{prox}}}}-\left(\frac{{{{{\rm{X}}}}}_{1}}{{{{{\rm{X}}}}}_{2}}\right)* ({{\it{D}}}_{{{\rm{prox}}}}-\,{{\it{D}}}_{{{\rm{dis}}}})}$$

where PDS is calculated by interpolation *D*_Ref_ with reference vessel diameter proximal to the stenosis (*D*_prox_), reference vessel diameter distal to the stenosis (*D*_dis_), distance between proximal and distal reference sites (X_1_), and distance between proximal reference site and stenosis site (X_2_) (Fig. 3).

*In vivo*, the readers also graded plaque types in the stenosis area into calcified, mixed, and noncalcified plaques as previously described [25].

### Statistical analysis

Statistical analysis was performed using SPSS (Version 29; IBM Corporation) and GraphPad (Version 8.4.2; GraphPad). The Shapiro–Wilk test was used to test for normal distribution of continuous variables, which were subsequently reported as means with standard deviations or medians with interquartile ranges. Categorial variables are reported as absolute frequencies and respective proportions. Cumulatively averaged deviations between *in vitro* measurements and ground-truth reference are calculated and presented in percent. Different image reconstructions were compared using the Wilcoxon related-samples signed rank test. A two-sided *p* < 0.050 was considered statistically significant. Inter- and intraobserver agreement was evaluated by Cohen’s κ and two-way random effects model intraclass correlation coefficients (ICC), using the following interpretation: < 0.5 poor, 0.5–0.75 moderate, 0.76–0.9 strong, and > 0.9 excellent agreement [26].

## Results

### *In vitro* assessment

**Fig. 4:**
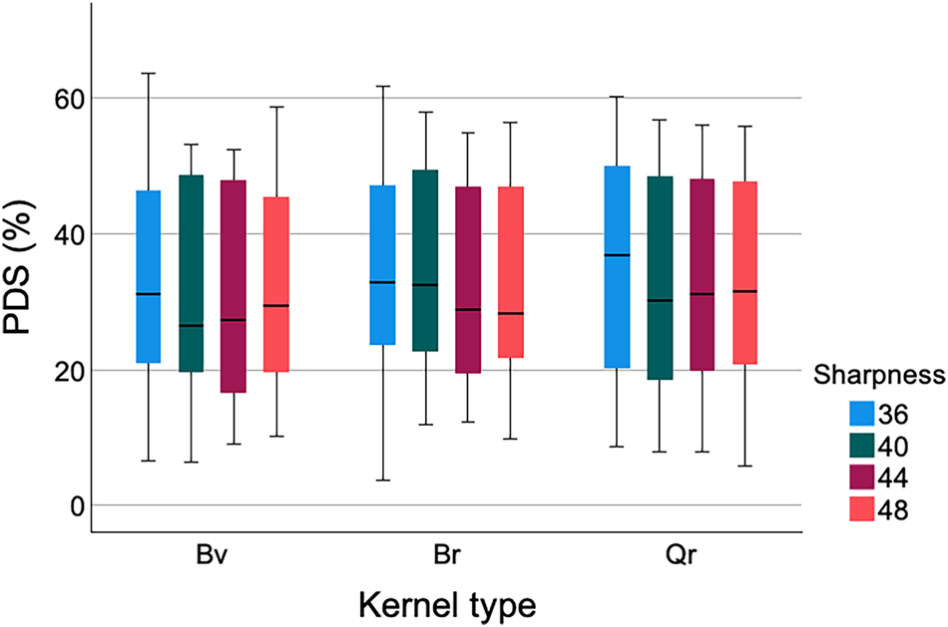
Overall evaluation *in vitro*. Overall evaluation across all stenoses in the three artificial vessels. Boxplots show PDS value, measured in reconstructions with different kernel types and sharpness levels

In the overall evaluation across all stenoses at different simulated heart rates, there was no difference in PDS measurements among reconstructions with different kernel types and sharpness levels (*p* = 0.412 to 0.990, Fig. 4). In the vessels-based analysis, a significant difference in measured PDS was observed in the vessels with calcified plaques, but not in the vessels with mixed plaques.

**Fig. 5:**
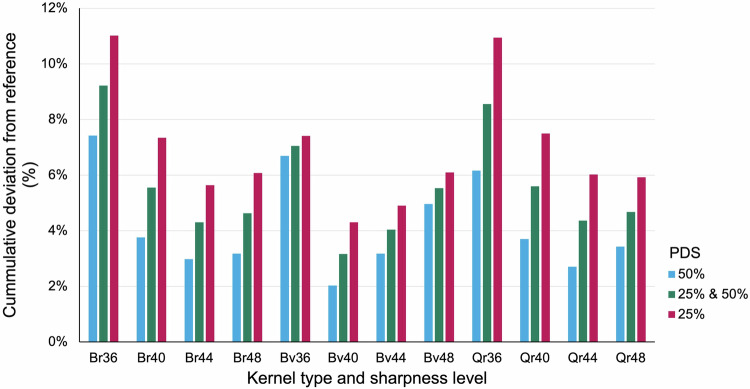
Cumulative deviation *in vitro*. Cumulative deviation of the measured PDS *in vitro* from the physical reference value in reconstructions with different kernel types and sharpness levels

Based on cumulative deviation, PDS was closer to the ground-truth reference standard in reconstructions with kernel Bv40 among all reconstruction kernels and sharpness levels, regardless of stenosis grade or plaque composition (Fig. 5).

In the *in vitro* measurement, the intraobserver reliability was excellent (ICC ≥ 0.99).

### Patient characteristics

A total of 34 patients were initially considered for inclusion in the study. Patients were excluded for non-evaluable coronary arteries (*n* = 2) and contraindication to nitroglycerin (*n* = 2). A total of 30 patients (8/30 [27%] females) with a mean age of 63 ± 13 years (range 39–83 years) and a mean body mass index of 25.4 ± 2.98 kg/m^2^ were included in the final analysis. The mean heart rate at the time of CT scan was 59 ± 7 beats per minute (Table 1). The mean effective radiation dose was 1.07 ± 0.18 mSv.

**Table 1:** Patient characteristics (*n* = 30)

Age, years	63 ± 13
Sex, male/ female	22 (73%)/8 (27%)
Body mass index, kg/m^2^	25.4 ± 3.0
Heart rate, beats per minute	59 ± 7 (range 37–70)
*Radiation dose, mSv	1.07 ± 0.18
CAD-RADS 0	16/30 (54%)
CAD-RADS 1	4/30 (13%)
CAD-RADS 2	3/30 (10%)
CAD-RADS 3	1/30 (3%)
CAD-RADS 4	6/30 (20%)

Data are presented as mean ± standard deviation or *n* (%), unless otherwise specified

*CAD-RADS* Coronary artery disease-reporting and data system

* Effective dose in mSv was used for radiation dose

Of the 53 coronary stenoses that were identified in the study sample, the majority were caused by calcified plaques (31/53, 58.5%), whereas mixed plaques (14/53, 26.4%) and noncalcified plaques (8/53, 15.1%) were less frequent.

### *In vivo* stenosis assessment

**Fig. 6:**
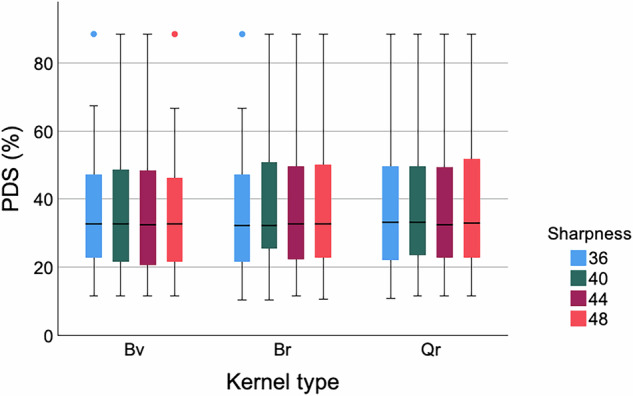
Overall evaluation *in vivo*. Boxplots show PDS values for *in vivo* stenoses, measured in different reconstructions with different kernel types and sharpness levels

In the per stenosis analysis, the overall median PDS showed no significant difference among the reconstructions with different kernel types and sharpness levels (all *p*-values Bv, Br, Qr = 1.000) (Fig. 6).

**Fig. 7:**
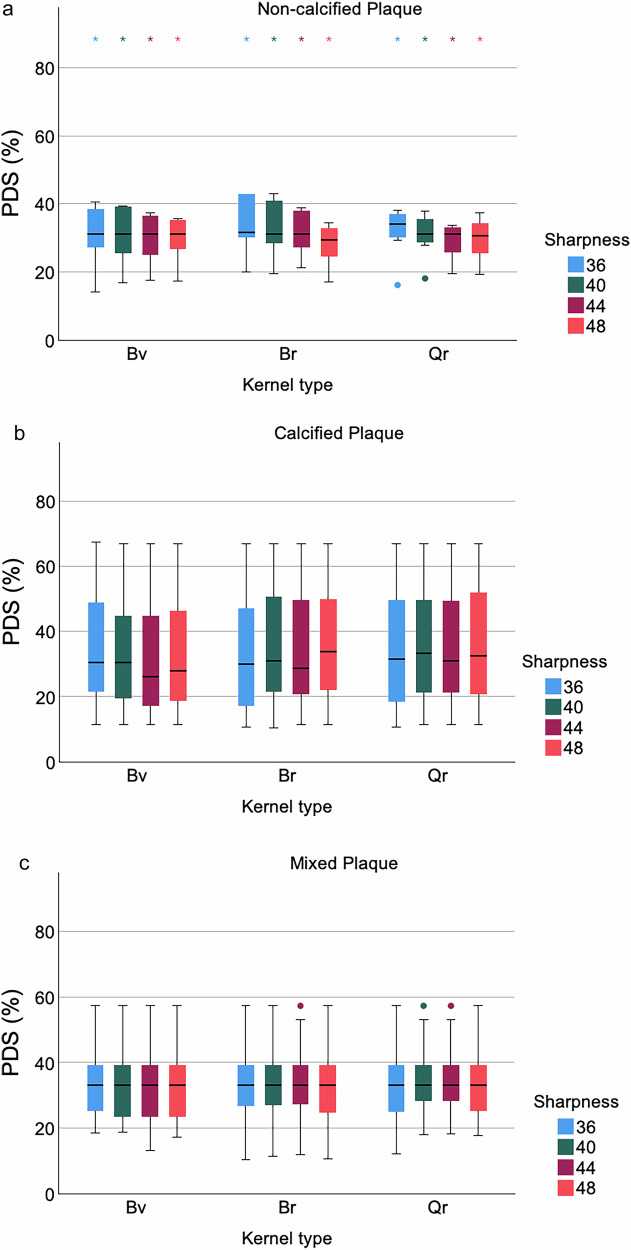
Evaluation with different plaque types *in vivo*. Boxplots show PDS values stratified by plaque type in reconstructions with different kernel types and sharpness levels: noncalcified, calcified, and mixed plaques (Fig. 7a–c, respectively).

When stratified by plaque type, only slight differences in PDS measurement were observed among different kernel types and sharpness levels, especially in calcified and noncalcified plaques. Overall, there was no evidence of a significant difference between plaque types (all *p*-values = 1.000) (Fig. 7).

The overall interobserver reliability was strong (ICC ≥ 0.88). Among different kernels, the highest interobserver reliability was found in the reconstructions with the Bv kernel (ICC_Bv_ ≥ 0.91, ICC_Qr_ ≥ 0.88, ICC_Br_ ≥ 0.85).

## Discussion

This *in vitro* and *in vivo* study investigated the impact of different reconstruction kernels and sharpness levels on the quantification of coronary artery stenosis in high-pitch PCD-CCTA. The main results are as follows: (1) in both *in vitro* and *in vivo* analysis, there was no significant difference in PDS measurements among different reconstruction kernels and sharpness levels; (2) in the *in vitro* analysis, PDS was measured closer to the ground-truth reference standard in reconstructions with kernel Bv40; and (3) in the *in vivo* study, the highest interobserver reliability for PDS measurements was observed in the reconstructions with the Bv kernels.

CCTA is considered a first-line test for the diagnosis and exclusion of coronary artery stenosis [2–4, 27]. Since the introduction of clinical PCD-CT, the image quality of CCTA has been further improved with increased sharpness, reducing blooming of calcifications, and leading to superior accuracy in stenosis quantification [11, 28]. However, as PCD-CT is a recently introduced technology, only limited literature is available regarding the effect of varying reconstruction parameters for coronary stenosis quantification. To our knowledge, no previous study has systematically evaluated the impact of the convolution kernel and kernel sharpness level on stenosis quantification in high-pitch PCD-CCTA.

In the recently published *in vivo* study, Augustin et al investigated the impact of kernel sharpness level (Bv36/48/56) on image quality, diagnostic confidence, and stenosis quantification in lower extremity PCD-CTA [29]. They found that changing kernel sharpness did not significantly affect the sensitivity and specificity of stenosis assessment. However, the diagnostic concordance of stenosis grading compared with DSA increased significantly with sharper kernels. While our study demonstrated no such observation, it should be noted that the lower extremity artery scans were performed using spiral acquisitions (*versus* the high-pitch scan mode in our study), and the lower extremity scans were reconstructed with a single vascular kernel. Furthermore, instead of being quantified, lower extremity vessel stenoses were graded on a four-point scale.

Mergen et al [19] reported the influence of kernel sharpness levels on image quality and blooming artefacts in patients with moderate to severe calcium deposits. In their study, PCD-CCTA was performed using the sequential UHR scan mode, and optimal quality for coronary plaque characterization and delineation of adjacent vessel lumen was found in reconstructions using kernel Bv64. Michael et al [30] investigated image quality for cardiac stents in reconstructions with different vascular kernels. In their phantom study, improved image quality to assess the in-stent lumen was found in reconstruction with kernel Bv72. In contrast to our study, CCTAs were performed in UHR scan mode in the above studies. In this scan mode, a higher sharpness level is preferable to exploit the potential of the spatial resolution given by the pixel size [19]. However, in high-pitch mode, the maximum spatial resolution is not directly defined by the pixel size since an interpolation of the raw data is required. This explains the preference for the lower sharpness level, Bv40, in high-pitch scan mode, as shown in our study results.

While the aforementioned studies analyzed the effect of various reconstruction parameters in sequential or UHR PCD-CTA, our study investigated the influence of different kernels and sharpness levels on stenosis quantification in spectral high-pitch PCD-CTA. In our study, there was no significant difference in PDS measurements between different kernels and sharpness levels. However, the highest interobserver reliability in *in vivo* stenosis assessment was found in reconstructions with the vascular (Bv) kernel, and *in vitro* PDS measurements with kernel Bv40 showed the value closest to the reference standard. Taken together with a previous study on image quality [18], our results indicate that kernel Bv40 may be the ideal reconstruction parameter for PCD-CCTA in high-pitch scan mode to achieve accurate stenosis quantification and excellent image quality.

As it is well known, CCTA is a first-line test for the evaluation of patients with suspected CAD, especially in patients with low and intermediate risk. The majority of this CCTA population were patients without relevant stenosis requiring further downstream testing or intervention [31]. Although several studies have reported the benefits of UHR CCTA in reducing calcium blooming and superior accuracy in quantifying coronary stenosis [19, 28, 32–35], there are still some advantages of the high-pitch scan mode. Compared with UHR scan mode, which is likely to suffer from lower *z*-coverage and longer scan duration and may be susceptible to respiratory motion and heart rate variability [19, 33], PCD-CCTA with high-pitch scan mode provides excellent image quality with significantly shorter acquisition time and lower radiation dose [36, 37]. With increasing image quality due to advanced temporal resolution in the third-generation dual-source CT [38] and the higher photon efficiency of PCD [39], the threshold above which heart rate begins to affect diagnostic quality is also increasing in PCD-CCTA with high-pitch scan mode [40–42]. Considering the above aspects, our result is particularly useful for patients with low and intermediate pretest probabilities and patients with low coronary calcium burden, who need CCTA to exclude significant CAD.

The following limitations merit consideration. First, in the *in vitro* study, only two stenosis grades, two plaque types (calcified and mixed plaques), at three simulated heart rates were investigated. Second, the *in vivo* analysis included a limited number of patients undergoing PCD-CCTA to exclude or detect CAD in a single center. Third, there was no physical reference for *in vivo* measurements, since almost all patients were not indicated for subsequent invasive coronary angiography. Finally, in this study, we investigated only the impact of different kernels and sharpness levels on coronary artery stenosis quantification. The effects of other reconstruction parameters, such as quantum iterative reconstruction or keV levels, should be evaluated in future studies.

In conclusion, image reconstruction with different kernel types and with sharpness levels between 36 and 48 can be used in high-pitch CCTA on PCD-CT with comparable diagnostic accuracy. Due to its better interobserver reliability *in vivo* and its closest measurements to the ground-truth reference *in vitro*, together with the results of a previous study on image quality, reconstruction with the Bv40 kernel should be preferred.

## Data Availability

The datasets used and/or analyzed during the current study are available from the corresponding author on reasonable request.

## Abbreviations

Br: Body regular
Bv: Body vascular
CAD: Coronary artery disease
CCTA: Coronary computed tomography angiography
CT: Computed tomography
PCD: Photon-counting detector
PDS: Percentage diameter stenosis
Qr: Quantitative regular
UHR: Ultra-high-resolution
